# 

**Published:** 1878-11

**Authors:** 


### CONTENTS.

ORIGINAL.

ART. I.—Scirrhus of the Pancreas. By J. F. Waid M. D., .... 121

ART. II.—Abstract of the Proceedings of the Buffalo Medical Association, .... 124

ART. III.—Asphalt, where obtained and how used. By * * * M. D., .... 128

MISCELLANEOUS.

Paracentesis Thoracis .... 130

Chloramyl, a new Anæsthetic and an improved Inhaler, .... 140

Annual Meeting of the American Gynæcological Society, .... 142

To a Young Doctor, from his Father, (A Homœopathic Physician, .... 149

EDITORIAL.

Libel Suit against the Editor of the American Journal of Insanity, .... 151

Dr. C. Henri Leonard's Books, .... 152

Appointments to Utica Insane Asylum, .... 152

Antiseptic Atomiser .... 153

Circular from Surgeon-General to Physicians and others, .... 153

Items .... 154

"Eye and Ear Infirmary," or Occulist and Aurist, by way of Advertisement .... 155

Important Medical Works for Publication, .... 155

BOOKS REVIEWED:

Fownes' Manual of Chemistry, .... 156

Barnes on Diseases of Women, .... 156

Nervous Diseases. By Allan McLane Hamilton, M. D. .... 158

Books and Pamphlets Received, .... 160

